# Supplementary material for: Revealing architectural order with quantitative label-free imaging and deep learning
Source: eLife. 2020 Jul 27;9:e55502. doi: 10.7554/eLife.55502 (PMC7431134; doi:10.7554/eLife.55502)
Supplement: Transparent reporting form [file elife-55502-transrepform.docx]

***eLife’s* transparent reporting form**

We encourage authors to provide detailed information *within their submission* to facilitate the interpretation and replication of experiments. Authors can upload supporting documentation to indicate the use of appropriate reporting guidelines for health-related research (see [EQUATOR Network](about:blank)), life science research (see the BioSharing Information Resource), or the [ARRIVE guidelines](about:blank) for reporting work involving animal research. Where applicable, authors should refer to any relevant reporting standards documents in this form.

If you have any questions, please consult our Journal Policies and/or contact us: [editorial@elifesciences.org](about:blank).

**Sample-size estimation**

- You should state whether an appropriate sample size was computed when the study was being designed
- You should state the statistical method of sample size computation and any required assumptions
- If no explicit power analysis was used, you should describe how you decided what sample (replicate) size (number) to use

Please outline where this information can be found within the submission (e.g., sections or figure legends), or explain why this information doesn’t apply to your submission:

We do not evaluate a biological hypothesis in this paper. Therefore, statistical methods of estimating sample size required to test a hypothesis are not applicable.

However, we report supervised deep learning models that are trained with the statistical optimization method of stochastic gradient descent. Our models translate label-free images into fluorescence images.

Accurate and generalizable models require large enough sample size that captures dynamic range of density, anisotropy, and myelination as well as morphological diversity of these measurements. We imaged whole brain tissue slice during a given acquisition. We kept pooling data from different tissue slices until the model yielded Pearson correlation coefficients and structural similarity index (SSIM) of ~0.8 between the target myelination and predicted myelination in the test set. The test set consisted of fields of view from both developmental time points and was not used for training or validation of the model. We employed the same approach to select sample size for various U-Net models of F-actin and nuclei for kidney tissue.

The metrics of accuracy are shown in Tables. The sample sizes used for training, validation, and test are described in “Image acquisition and registration” and “Model training and inference” in Methods.

**Replicates**

- You should report how often each experiment was performed
- You should include a definition of biological versus technical replication
- The data obtained should be provided and sufficient information should be provided to indicate the number of independent biological and/or technical replicates
- If you encountered any outliers, you should describe how these were handled
- Criteria for exclusion/inclusion of data should be clearly stated
- High-throughput sequence data should be uploaded before submission, with a private link for reviewers provided (these are available from both GEO and ArrayExpress)

Please outline where this information can be found within the submission (e.g., sections or figure legends), or explain why this information doesn’t apply to your submission:

Number of biological human brain slice replicates are described in the section titled “Predicting myelination in sections of developing human brain”. For training models, human brain images were curated to exclude images with staining artifacts (as described in Results and Methods).

Each field of view of the acquisition represents an independent measure of the random noise associated with protocols, equipment, and stochastic learning. Thus, number of technical replicates is equal to number of fields of view used for training, validation, and test, which are 168 + 35 for GW24; 210 + 76 for GW20. The human brain dataset has 4 biological replicates, 2 for GW20 and 2 for GW24.

Since the kidney tissue data was acquired to develop various neural network architectures, rather than analyze specific structure, we did not acquire and analyze a biological replicate of Kidney tissue. The number of technical replicates is 160, from which few fields of view were excluded to avoid staining artifacts.

**Statistical reporting**

- Statistical analysis methods should be described and justified
- Raw data should be presented in figures whenever informative to do so (typically when N per group is less than 10)
- For each experiment, you should identify the statistical tests used, exact values of N, definitions of center, methods of multiple test correction, and dispersion and precision measures (e.g., mean, median, SD, SEM, confidence intervals; and, for the major substantive results, a measure of effect size (e.g., Pearson's r, Cohen's d)
- Report exact p-values wherever possible alongside the summary statistics and 95% confidence intervals. These should be reported for all key questions and not only when the p-value is less than 0.05.

Please outline where this information can be found within the submission (e.g., sections or figure legends), or explain why this information doesn’t apply to your submission:

We describe metrics, Pearson correlation and structural similarity index (SSIM), for quantifying predicted image quality in “Model evaluation” in Methods. We don’t propose a biological hypothesis and hence other statistical analysis is not applicable to the paper.

(For large datasets, or papers with a very large number of statistical tests, you may upload a single table file with tests, Ns, etc., with reference to sections in the manuscript.)

**Group allocation**

- Indicate how samples were allocated into experimental groups (in the case of clinical studies, please specify allocation to treatment method); if randomization was used, please also state if restricted randomization was applied
- Indicate if masking was used during group allocation, data collection and/or data analysis

Please outline where this information can be found within the submission (e.g., sections or figure legends), or explain why this information doesn’t apply to your submission:

Images (samples) were randomly split into training, validation, and test sets as described in “Model training and inference” in Methods.

**Additional data files (“source data”)**

- We encourage you to upload relevant additional data files, such as numerical data that are represented as a graph in a figure, or as a summary table
- Where provided, these should be in the most useful format, and they can be uploaded as “Source data” files linked to a main figure or table
- Include model definition files including the full list of parameters used
- Include code used for data analysis (e.g., R, MatLab)
- Avoid stating that data files are “available upon request”

Please indicate the figures or tables for which source data files have been provided:

Python software for image reconstruction and raw microscope images of examples shown in Fig. 2 are available for download at [https://github.com/mehta-lab/reconstruct-order](about:blank).

The raw image data is hosted on google drive and can be fetched using the functions in the above repository.

Python software for training the neural networks and inference is available at: [https://github.com/czbiohub/microDL](about:blank).

The datasets that enable trainining of image translation models are hosted on BioImage Archive:

<https://www.ebi.ac.uk/biostudies/BioImages/studies/S-BIAD25>
